# A Clinical Study of Tracheobronchopathia Osteochondroplastica: Findings from a Large Chinese Cohort

**DOI:** 10.1371/journal.pone.0102068

**Published:** 2014-07-11

**Authors:** Ying Zhu, Ning Wu, Hai-Dong Huang, Yu-Chao Dong, Qin-Ying Sun, Wei Zhang, Qin Wang, Qiang Li

**Affiliations:** Department of Respiratory Medicine, Changhai Hospital, Second Military Medical University, Shanghai, China; University of Leuven, Rega Institute, Belgium

## Abstract

**Background and Study Aims:**

Tracheobronchopathia osteochondroplastica (TO) is an uncommon disease of the tracheobronchial system that leads to narrowing of the airway lumen from cartilaginous and/or osseous submucosal nodules. The aim of this study is to perform a detailed review of this rare disease in a large cohort of patients with TO proven by fiberoptic bronchoscopy from China.

**Patients and Methods:**

Retrospective chart review was performed on 41,600 patients who underwent bronchoscopy in the Department of Respiratory Medicine of Changhai Hospital between January 2005 and December 2012. Cases of TO were identified based on characteristic features during bronchoscopic examination.

**Results:**

22 cases of bronchoscopic TO were identified. Among whom one-half were male and the mean age was 47.45±10.91 years old. The most frequent symptoms at presentation were chronic cough (n = 14) and increased sputum production (n = 10). Radiographic abnormalities were observed in 3/18 patients and findings on computed tomography consistent with TO such as beaded intraluminal calcifications and/or increased luminal thickenings were observed in 18/22 patients. Patients were classified into the following categories based on the severity of bronchoscopic findings: Stage I (n = 2), Stage II (n = 6) and Stage III (n = 14). The result that bronchoscopic improvement was observed in 2 patients administered with inhaled corticosteroids suggested that resolution of this disease is possible.

**Conclusions:**

TO is a benign disease with slow progression, which could be roughly divided into 3 stages on the basis of the characteristic endoscopic features and histopathologic findings. Chronic inflammation was thought to be more important than the other existing plausible hypotheses in the course of TO. Inhaled corticosteroids might have some impact on patients at Stage I/II.

## Introduction

Tracheobronchopathia osteochondroplastica (TO) is a rare benign disorder of unknown etiology, characterized by the presence of multiple cartilaginous and/or osseous submucosal nodules protruding into the lumen of lower two thirds of the trachea and upper part of the main bronchi [1]. In some cases, larynx and subglottic trachea were also involved [2], [3]. The posterior membranous wall of the tracheal is typically spared, and the overlying mucosa is intact with normal or metaplastic epithelium [4]. TO was previously an incidental finding at autopsy due to its usually benign progression and lack of typical symptoms. It is presently increasingly discovered through bronchoscopy and/or chest computed tomography (CT) [5].

Most published papers on TO referred to a single case or small series. The largest multicentral study reported in 2001 involved 41 cases [6]. However, most of what we know about the disease is from Western literatures and this is the first large study of 22 cases from China. The etiology of TO is still unclear and there’s no standard treatment guideline. In this retrospective study, we tried to provide a better description of the whole picture of TO, including the most probable etiology and appropriate medical management based on our findings.

## Methods

Among the 41600 patients who had undergone fiberoptic bronchoscopies in the Department of Respiratory Medicine of Changhai Hospital between January 2005 and December 2012, 22 were confirmed of TO by endoscopy with or without histopathologic findings. The characteristic manifestations were defined as multiple papilla-like sessile cartilaginous or bony nodules, arising from the submucosa and protruding selectively from the anterolateral wall of the tracheobronchial tree [7]. This retrospective study has been approved by the Institutional Review Board of Changhai Hospital. Patient consents were not needed because of the retrospective nature, but all the patients had assigned the informed consent form before they underwent bronchoscopic examination. These patient records were anonymized and de-identified prior to analyses.

Medical records of these patients including demographics, clinical presentations, bronchoscopic and histopathologic features, managements and outcomes were retrospectively analyzed. All patients were followed up every 3–6 months for at least 3 years.

## Results

Among the 41600 patients, 22 (male, 11; overall rate, 0.05%) were identified as having TO. As shown in Table 1, the age ranged from 20 to 63 years old (mean, 47.45±10.91 yr). Of the 22 patients, 8 had a history of chronic or recurrent infectious diseases of tracheobronchus, 12 had a long-term exposure to dust or irritant gases due to occupational factors, and 4 were active or former smokers. One patient suffered from lung squamous carcinoma concomitantly. These data showed that chronic inflammation may be an important etiology.

**Table 1:** Patients information of TO.

Patient	Age, Sex	Localization	Symptoms	Chest X-rayabnormality	CT scanfindings	BronchoscopicClassification(Type, Stage)	Treatment	Outcome
1	50, F	Lower 2/3 ofthe trachea	Cough	Normal	Irregular thickening,calcification	Scattered, II	InhaledCorticosteroids	Symptoms relieved
2	54, F	Trachea and leftmain bronchi	Cough andexpectoration	Normal	-	Scattered, I	InhaledCorticosteroids	Symptoms relieved
3	37, M	Trachea andbilateral main bronchi	Cough, expectoration	Normal	Calcification	Diffuse, II	InhaledCorticosteroids	Significantlyimproved bronchoscopically
4	37, F	Trachea andbilateral main bronchi	Cough andexpectoration and dyspnea	Normal	Irregular thickening,and tracheal stenosis	Scattered, III	Symptomatictreatment	Symptoms relieved, dyspneaoccasionally
5	45, M	Trachea andbilateral main bronchi	Cough	Normal	-	Diffuse, II	InhaledCorticosteroids	Significantly improved bronchoscopically
6	50, F	Trachea and rightmain bronchi	Cough anddyspnea	Normal	Irregular thickening,calcification and trachealstenosis	Diffuse, III	Symptomatictreatment	Symptoms relieved, dyspneaoccasionally
7	60, M	Trachea andbilateral main bronchi	Dyspnea, chesttightness and pain	Pleural effusion	Irregular thickening,calcification	Diffuse, III	Symptomatictreatment	Died (as a result of primary malignancy)
8	62, F	Trachea andbilateral main bronchi	Asymptomatic	Normal	-	Scattered, II	InhaledCorticosteroids	Asymptomatic(stable bronchoscopically)
9	56, F	Upper trachea	Cough andexpectoration	Normal	Irregular thickening	Scattered, II	InhaledCorticosteroids	Symptomsrelieved
10	40, F	Trachea andbilateral main bronchi	Cough, expectoration	-	Irregular thickening	Scattered, II	InhaledCorticosteroids	Symptomsrelieved
11	48, M	Trachea andbilateral main bronchi	Cough andexpectoration	Infiltrate	Irregular thickening,calcification andtracheal stenosis	Confluent, III	Symptomatictreatment	Symptomsrelieved
12	58, F	Trachea andbilateral main bronchiRight intermedius bronchus,Left lingular bronchus	Chest tightnessand pain	Normal	Irregular thickening,calcification, trachealand bronchial stenosis	Diffuse, III	Symptomatictreatment	Symptoms relieved
13	55, M	Lower 2/3 ofthe trachea	Dyspnea and chesttightness and pain	Infiltrate	Irregular thickening,calcification	Diffuse, III	Symptomatictreatment	Symptoms relieved, dyspneaoccasionally
14	36, M	Infraglottic portion,trachea and bilateralmain bronchi	Dyspnea, chesttightness and pain	Normal	Irregular thickening,calcification	Confluent, III	Symptomatictreatment	Symptoms relieved, dyspneaoccasionally
15	59, F	Trachea andbilateral main bronchi	Dryness ofthe throat	Normal	-	Scattered, I	InhaledCorticosteroids	Symptoms relieved
16	30, F	Trachea andbilateral main bronchi	Cough	-	Irregular thickening,calcification	Diffuse, III	Symptomatictreatment	Symptoms relieved
17	41, M	Trachea andright main bronchi	Cough, expectorationand weakness	Normal	Irregular thickening,calcification andtracheal stenosis	Diffuse, III	Symptomatictreatment	Symptoms relieved
18	43, M	Lower 2/3 ofthe trachea	Expectoration and drynessof the throat	Normal	Irregular thickening,calcification	Scattered, III	Symptomatictreatment	Symptomsrelieved slightly
19	46, M	Upper trachea	Cough andexpectoration	Normal	Irregular thickening,calcification	Confluent, III	Symptomatictreatment	Symptoms relieved
20	20, M	Trachea andbilateral main bronchi	Cough and fever	Normal	Irregular thickening,calcification	Confluent, III	Symptomatictreatment	Symptomsrelieved
21	63, F	Trachea andbilateral main bronchi	Cough, expectorationand fever	Normal	Irregular thickening,calcification andtracheal stenosis	Diffuse, III	Symptomatictreatment	Symptomsrelieved
22	54, M	Trachea andbilateral main bronchi	Hoarseness	-	Irregular thickening	Diffuse, III	Symptomatictreatment	unchanged

Chronic coughs were the most common symptoms (14/22). Other clinical manifestations included sputum production (10/22), dyspnea on exertion (5/22), chest tightness and pain (4/22), continuous or intermittent fever (2/22), dryness of the throat (2/22), hoarseness (1/22) and weakness (1/22). Wheezing and stridor were found in 2 patients. Moreover, one patient was asymptomatic.

Chest radiography were performed in 18 patients and 15 (15/18) of them were negative. Pleural effusion related to metastatic cancer was observed in one patient, and pneumonia in two. CT scans were available for all patients and the common feature were presented with a “beaded” or scalloped appearance on the wall of airways, including irregular thickenings (17/22), calcifications (14/22), tracheal and bronchial stenoses (6/22 and 1/22 respectively) [8]. Calcification in left hilar lymph node was observed in one patient.

Characteristic bronchoscopic images were found in all of the 22 patients. The whole trachea was involved in 17 patients, upon upper trachea in 2 and lower two thirds of the trachea in 3 patients. The right main bronchus was slightly more frequently involved than the left part, in 16 and 15 patients, respectively. In two patients (2/22), the protrusions were disclosed in lobar and segmental bronchi. Infraglottic involvement was found in one patient. The categories of lesions were assessed as diffuse (10/22), scattered (8/22) and confluent type (4/22) under bronchoscopy, according to Dutau H et al [9].

Histopathologic results were achieved in 18 patients, which made the documentation of TO with typical cartilaginous and bone islands (11/18 and 14/18, respectively). Other frequent findings consisted of squamous metaplasia of the tracheal epithelium (14/18) and inflammatory cells infiltrations (6/18).

**Figure 1:**
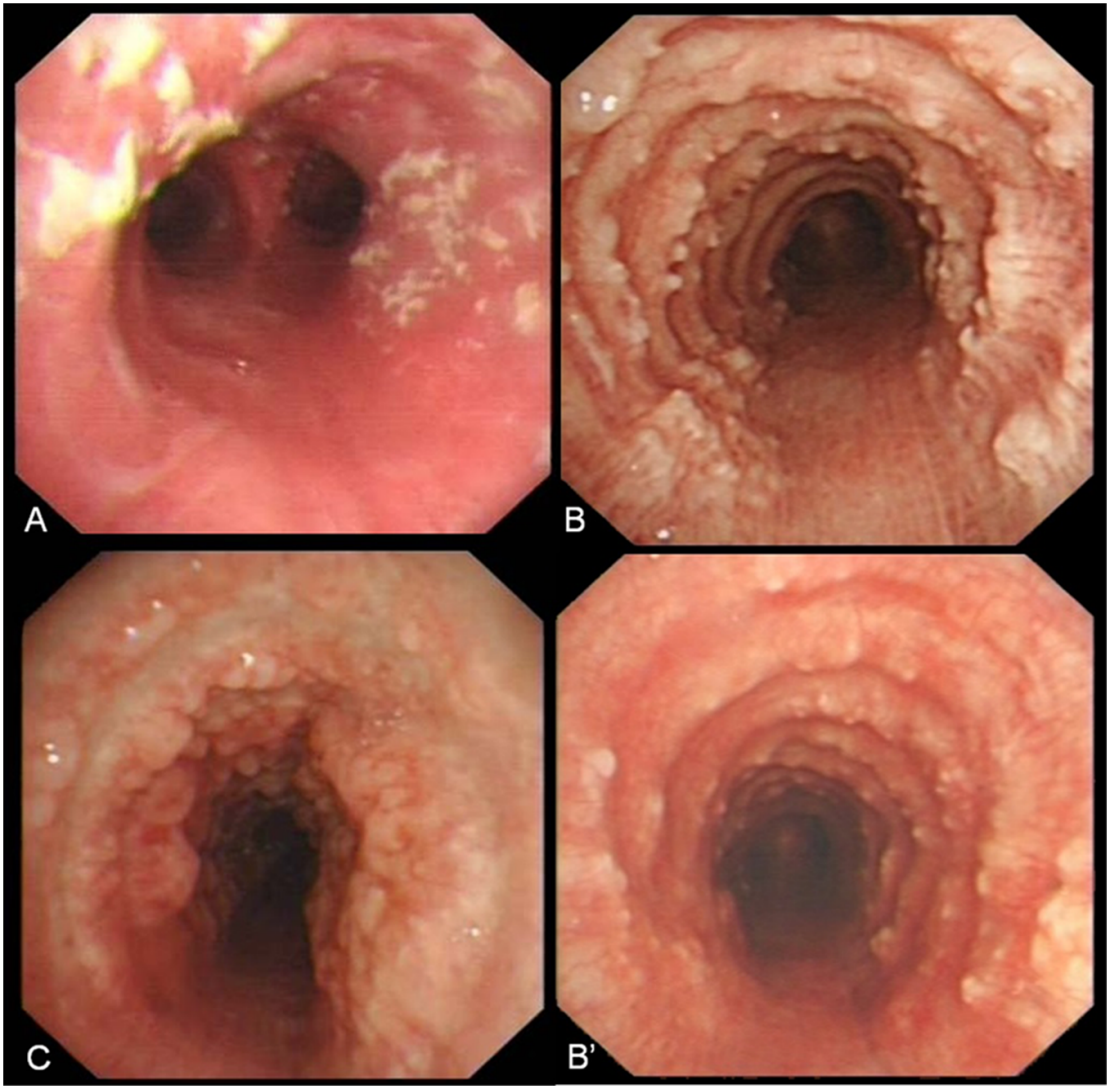
Characteristic bronchoscopic manifestations of TO. (A) Stage I: Scattered plaque-like inflammatory infiltrations of yellow-whitish soft lesions distributed overlying the mucosa of the lumen, accompanied with the change of mucosa hyperemia edema. (B) Stage II: Numerous dispersed or diffuse existence of both sessile spicules and cartilaginous nodules projecting into the lumen with a typical “cobblestone or stalactitic cave” visualization. (C) Stage III: A deformed, rigid and narrow feature of the airway, causing airway narrowness even obstruction. (B’) The same patient with Fig. 1B: The number of diffuse cartilaginous nodules decreased after one year treatment with inhaled budesonide.

According to the characteristic bronchoscopic visualization and histopathologic results, TO could be divided into three stages: Stage I (early stage, also a mild grade), Stage II (middle stage, moderate grade) and Stage III (late stage, severe grade). The bronchoscopic image of Stage I revealed that scattered plaque-like infiltrations of yellow-whitish soft lesions were distributed overlying the mucosa of the lumen, accompanied with a number of inflammatory cells and occasionally cartilaginous cells. (Figure 1A). The presentation of Stage II demonstrated dispersed or diffuse existence of both cartilaginous nodules and sessile spicules projecting into the lumen, which was often described as cobblestone or stalactitic cave. (Figure 1B). By contrast to the former two stages, Stage III was a severer grade, which showed the feature of a deformed and rigid tracheal wall, causing airway narrowness even obstruction (Figure 1C). Lamellated bone and fatty marrow with hematopoiesis could be seen in the bone protrusions of patients at this stage. As per this classification, group of Stage III, including 14 patients, was the largest group in this study, followed by Stage II (6/22) and Stage I (2/22), respectively.

Symptomatic treatments were performed including antitussive, expectorant, and antibiotics. Inhaled corticosteroids (budesonide power, 400 ug, bid) were administered to 8 patients, two of whom belong to Stage I and six to Stage II. Chemotherapy and antibiotics were performed in patients with coexisting cancer and pneumonia, respectively. No patient underwent any bronchoscopic intervention, stent replacement or surgical resection. The average follow-up duration was 5.36±2.10 yrs (range, 3–8 yrs). Nineteen patients (19/22) had a remission of symptoms to some extent while their bronchoscopic presentations remained unchanged. Remarkable improvement of the lesions under endoscopy was observed in 2 patients treated with inhaled corticosteroids 1 year later (2/8) (Figure 1B’). One patient (1/22) asymptomatic kept stable bronchoscopically. One patient (1/22) remained hoarseness and one (1/22) died of exacerbation of lung cancer 2 years later.

## Discussion

TO was originally described in 1857 by Wilks who referred to autopsy findings of a number of bony plates in a 38-year-old man died of tuberculosis [10], [11]. In 1896, Von Schroetter documented the diagnosis in vivo for the first time by using a laryngeal mirror [12]. It was not until 1897 that the earliest bronchoscopic description of TO was firmly given by Killian. The term of ‘tracheopathia osteoplastica’ was proposed by Aschoff in 1910 and expanded by Landsberg as ‘tracheobronchopathia osteochondroplastica’ [13], [14]. Since then, cases of TO have been reported increasingly. To our knowledge, up to now, more than 500 cases of TO have been reported worldwide, including approximately 140 cases in Japanese literatures and 80 in Chinese publications [15], [16]. However, the true incidence of TO must be a lot higher owing to its absent or nonspecific symptomatic nature. It has been estimated that only half of the people with TO could be recognized in their lifetime [17]. The detection rate of this disease ranged from 1∶400 (0.25%) to 3∶1000 (0.30%) in autopsies and 1∶125 (0.80%) to 1∶10000 (0.01%) via bronchoscopy [1], [18]–[20]. In our study, 22 of 41600 patients (0.05%) undergone bronchoscopy were confirmed as TO. The mean age of our TO patients was around mid-50s, and there was no difference in gender distribution, as described elsewhere [3], [13], [16], [21]–[25].

The etiology of TO remains unknown. Numerous presumptive theories have been proposed such as ozaena, genetic factor and inheritance, tissular degenerative process, metabolic disturbance, calcium and phosphorus metabolic disorders, congenital anomaly, chemical or mechanical irritation, primary amyloidosis and malignancy [3], [25]–[31]. Among those, chronic inflammation was an important one, which was first suggested by Wilks in 1857. In a series of 15 cases with TO, all of the patients suffered from recurrent respiratory infections [32]. In our study, 8 patients had a history of chronic or recurrent infection of tracheobronchial diseases, 12 experienced a long time exposure of dust or irritate gases and 4 were active or former smokers. Furthermore, histopathologic results demonstrated a large number of inflammatory cells infiltration in the lesions of patients at early stage. It was estimated that a slowly progressing inflammatory process of the mucosa caused squamous metaplasia of the epithelium, damaged the normal architecture of the airway, affected the defense mechanisms especially the efficiency of mucociliary clearing respiratory secretions and led to recurrent infections at last [33]. Therefore, we suppose that chronic inflammation may cause collapse of normal defensive system and it might be a main factor in the development of TO. High incidence of TO at the cold climate region was reported in Finland and Northern Sweden. The unfavourable climate was regarded as an inclinded reason of chronic infection, causing the cold-air-related hyperreactivity of the airway epithelium [10]. However, the detailed pathogenesis is not clear. Ecchondrosis and exostosis put forth by Virchow in 1869 and metaplasia of the elastic tissue by Aschoff in 1910 were two main theories adopted in numerous publications [14]. Recently, bone morphogenetic protein-2 (BMP-2) and transforming growth factor beta-1 (TGF-β1) were considered the potent inducers for new bone formation [1].

According to the previous reviews, the most common clinical manifestations of TO were chronic cough and haemoptysis [2], [34]. In our series, chronic cough was also the most frequent respiratory symptom, but haemoptysis had not been observed. The positive rate of CT scan in our study was 81.82% (18/22) and 16.67% (3/18) of X-ray, which made CT a more important imaging modality recommended for patients suspected of having TO [35], [36]. In addition, CT scan is also useful in the following-up period.

Bronchoscopy remains the gold standard for recognition of TO [37], [38]. The typical bronchoscopic findings were described vividly as a cobblestone, a beaded or stalactitic cave, a rock garden, or a veritable mountainscape appearance [26], [39]. Biopsy of TO lesions sometimes can be a challenge due to the hard consistency of the bone nodules [26]. It was reported that histopathologic examination of TO nodules were usually unnecessary [40]. Nevertheless, because of unfamiliarity for many bronchoscopists with this condition and the need to exclude differential diagnoses, the histopathologic confirmation should be essential and reasonable in most cases [37]. We found a series of dynamic changes in the course of TO based on endoscopic features and histopathologic results, so three stages of TO were established. The performance at the early course of TO was presented with plaque-like inflammatory infiltrations, which could be neglected by most bronchoscopists and the treatment would be delayed. With the persistent stimuli and a long-term exposure of injured airway mucosa in the chronic inflammatory circumstance, the defensive system was impaired and cartilaginous nodules and/or bony spicules increasingly formed (Stage II), which was a relatively intermediate status lasting for several years and turned into the next classification. Histologic findings gave a strong evidence that prominent nodules formed in the portions where is absence of normal ciliated respiratory epithelium [41]. Patients at Stage III–the most serious stage of TO, were always accompanied with abnormal airway structure, leading to aggravating symptoms. In our study, the number of patients at this stage accounts for a large scale with 14 cases at the time of diagnosis. Five patients in this group appeared dyspnea and symptoms of others were severer than those at Stage I and Stage II.

The treatment of TO varies from symptomatic management, bronchoscopic intervention to operative correction depending on the severity of airway obstruction [2]. It has been suggested that inhaled corticosteroids may have some impact on the condition full of chondrocytes and inflammatory cells cited by Martin [42]. In Hinrich W’s report, one patient with TO suffered from chronic cough with haemoptysis recovered after receiving inhaled budesonide (200 ug, bid) [43]. Li SY et al reported one case whose symptoms and mucosal hyperemia were improved after treated with beclomethasone dipropionate and theophylline for 6 months [44]. In addition, Xie BS et al has proposed that symptoms of three patients with productive cough were ameliorated, imaging findings of pulmonary consolidation significant improved and cartilaginous nodules absorbed bronchoscopically after receiving budesonide, while there was no effect at the bone formation place [45]. Based on the endoscopic and histopathologic findings in our patients and above literatures, we assumed that inhaled corticosteroids could be useful to lesions filled with inflammatory cells and cartilaginous nodules, while it may have little impact on the lesions full of osseous cells and lamellated bone. Eight patients at Stage I/II were administered with inhaled budesonide. It turned out that the number of lesions with diffuse cartilaginous nodules decreased significantly in 2 patients after a 1-year treatment and the symptoms of other 6 patients were alleviated to a certain extent despite of little change under bronchoscopy. We speculated that inhaled corticosteroids could reverse the columnar cells and ciliated columnar epithelium, reduce airway basal cell from metaplasia and repair tissue partially [45]. Once lamellated bone appeared, there could be less chances of improving the bronchoscopic visualization. Nevertheless, appropriate dosage and therapeutic duration need further exploration. As for the patients at Stage III, they did not receive inhaled corticosteroids in our hospital. Apart from considering bone formation and decreased inflammatory cells, nearly half of the patients had taken the medication before they came to our department. But it turned out there was no effect on relieving their endoscopic findings or even symptoms. Besides, side effects such as oral mucosa ulcer, monilial infection and hoarseness appeared. Therefore these patients were administered with symptomatic treatment including antitussives, expectorants, antibiotics etc. Except one patient died of lung cancer and symptoms of one with hoarseness unchanged, the rest 12 patients remitted to some extent. Bronchoscopic correction and surgical approaches should be resorted to in the case of severe constriction in the lumen of central airways [15], [32], [46], [47].

## Conclusion

As per history diseases of our patients, chronic inflammation might be an important factor in the course of TO. Furthermore, lesions in the airway were filled with inflammatory cells, which make it possible that inhaled corticosteroids may have some impact on patients at the early two stages. We found most of the patients were at their third stage at the time of diagnosis in line with reported literatures. Hence, we proposed this staging system is to improve the cognition of physicians for this condition and give the medication to patients in time. We are attempting to reverse the condition and improve patients’ living quality. Whether inhaled corticosteroids should be used or helpful for patients at Stage III needs further study. More cases are needed to make out a more feasible treatment guideline.
